# A Study of Mental Health and Dyadic Adjustment Between Smokers and Nonsmokers

**DOI:** 10.5812/ijhrba.4175

**Published:** 2012-07-25

**Authors:** Bahman Kord Tamini, Mahvash Raghibi, Nour-Mohammad Bakhshani

**Affiliations:** 1 Department of Psychology, Faculty of Education and Psychology, University of Sistan and Baluchestan, Zahedan, IR Iran; 2 Department of Clinical Psychology, Zahedan University of Medical Sciences, Research Center for Children and Adolescents Health, Zahedan, IR Iran

**Keywords:** Mental Health, Psychiatric Status Rating Scales, Smoke

## Abstract

**Background:**

The mental health and dyadic adjustment of smokers is a matter of serious concern which brings many demerits on mental health as well as physical heath.

**Objectives:**

This study was performed to ascertain the relationship between mental health and dyadic adjustment of smokers and nonsmokers in Zahedan.

**Patients and Methods:**

The sample size consisted of 100 smokers and 100 nonsmokers selected through accessible sampling method. The General Health Questionnaire-28 (GHQ-28) and Dyadic Adjustment Scale (DAS) were used to collect the data. Pearson correlation, stepwise regression, and independent “*t*-test” were applied to analyze the data.

**Results:**

Results revealed that physical symptoms, anxiety, social dysfunction, and depression sub-scales, as well as total scores of mental health negatively correlated with dyadic adjustment. Stepwise regression showed the following results: in the total sample, physical symptoms accounted for 22.7% of the variance in dyadic adjustment; also in the total sample, physical symptoms and social dysfunction together explained 24.5% of the variance in dyadic adjustment; social dysfunction accounted for 30%, anxiety for 3.7%, depression for 7% and overall mental health for 3.5% of the variance in dyadic adjustment in the smokers’ sample. Results demonstrated that physical symptoms explained 15.9% of the variance in dyadic adjustment in the nonsmokers’ sample. Results further revealed that the mean scores of physical symptoms and anxiety sub-scales, and the total scores of the mental health of smokers were greater than those of nonsmokers. However, no significant differences appeared between the two groups on social dysfunction and depression. Finally, the research revealed that the mean scores of dyadic adjustment were greater for nonsmokers than for smokers.

**Conclusions:**

The research revealed that nonsmokers showed better mental health and dyadic adjustment than smokers, thus suggesting that smoking endangers and can impair the tranquility of families and that smoking also threatens the dyadic adjustment of couples.

## 1. Background

Smoking is the most common risk behavior engaged in by youth and young adults. Although smoking is a main contributing factor for cancer, coronary heart disease, lung disease, and other severe diseases, contemporary society cannot bring itself to deal directly with this issue. Indeed, the risk behavior of smoking is not prohibited by society even though evidence provided by extensive research demonstrates that not smoking is the most significant factor in mortality prevention (1, 2). Researchers have approached the issue of smoking in different ways (2). In the discipline of social psychology, investigators have concentrated on behavioral factors involved in smoking itself and in smoking cessation. Other researchers have focused their efforts on identifying the factors and behaviors that lead to smoking among youth and young adults (3). From the United States, cigarette smoking and other tobacco use has spread to other countries; besides that, tobacco consumption has increased over time. Studies show that in the 1920s, approximately 20% of men and 5% of women smoked, but in the 1980s, the rates had increased to 53% of men and 33% of women. At the same time, the daily amount, or mean, of smoking also increased: in 1935, daily consumption was 12 cigarettes; in 1959, daily consumption increased to 26 cigarettes; and in 1979, it increased to 33 cigarettes daily (2). Current estimates claim that, globally, one billion individuals smoke tobacco.

In the issue of tobacco use, however, the basic question is, “What are the factors that actually cause people to begin smoking and to continue smoking cigarettes? ”Existing reports assert that psychological factors are important aspects in the perception and understanding of smoking behaviors. According to Maher (2), the American Medical Council reports that smoking inception depends on peer and family behavior and that smoking cessation is related to social and psychological factors. More specifically, the Council’s report divides smoking behaviors and the hidden motives of smokers into four stages, as follows: 1) preparation, 2) inception, 3) being a smoker, and 4) maintaining as a smoker.

1) Preparation: Before a cigarette ever touches a potential smoker’s lips, that individual has already developed perceptions, attitudes, and personal beliefs about cigarettes and smoking through observation of smokers (especially parents) and mass media. The potential smoker creates a personal image of the qualities related to smoking cigarettes and what smoking indicates socially. Furthermore, three forms of attitudes may lead youths to begin smoking: 1) equilibrium imagination, 2) support from peers, and 3) desire to decrease tension and do tasks well.

2) Inception: In this most important stage, the inception of smoking happens in conformity with peer groups. Having smokers in the family, especially the father, accelerates this process (2).

3) Being a smoker: Studies indicate that it takes two years to become a Full-fledged smoker. Young smokers with impulsive behavior believe that smoking will not harm them and consequently increase their amount of smoking.

4) Maintaining as a smoker: In this last stage, biological mechanisms and psychological factors hold constant the behavioral patterns of smoking. The psychological effective factors of smoking are habituation, addiction, reduction of anxiety and stress, leisure, socialization, social reward, arousal, and motivation. The biological factors of smoking are nicotine’s reinforcement effect and the conditioned need to keep a standard level of nicotine in the blood (2).

Therefore, it may be deduced that psychological factors motivate individuals to light a first cigarette, and then biological, along with psychological, factors motivate its continuation. According to a World Health Organization Report (4),“Emotional (mood and anxiety) disorders and cigarette smoking are highly prevalent and co morbid”. Researchers have revealed a mutual association between emotional disorders and smoking; each may be a risk factor for the other (5). Results of some studies revealed a strong relationship between smoking and certain emotional disorders (6). Morrell (7, 8), for example, reported similar findings: nearly 41% of smokers reported receiving a mental health diagnosis within the previous month (9). According to Colton and Manderscheid (10), public mental-health clients have a higher relative risk of death than the general population due, in part, to high rates of tobacco use. Investigators revealed that among current smokers, the most common mental health diagnoses are the following: alcohol abuse, major depression, substance abuse, and anxiety disorders such as simple phobias and social phobias (9, 11). Other studies examined factors of anxiety, depression, smoking, and implementation of smoking-prevention programs among high school students. Results indicated that students who smoked had higher mean scores on anxiety symptoms and depression than students who did not smoke. Results also showed that students who smoked “just for fun” exhibited higher anxiety than nonsmokers. Sonia et al. (12) studied depression symptoms, smoking, drinking, and quality of life among patients with head and neck cancer. Findings showed that 46% of these patients had depression, and 30% of them smoked. Overall, researchers and practitioners have acknowledged a significant positive relationship between mental illness and smoking (13, 14). Surveys of public populations showed a significant relationship between smoking and current psychological illness (15, 16), and several other studies indicated that a high percentage of smokers have mental disorders (9, 17, 18), illustrating that individuals with psychological disorders are twice as likely to smoke than individuals without psychological disorders. Finally, another study (19) revealed that girls report smoking at a lower rate than boys, but age and positive family relationships were strongly associated with smoking in both genders. Researchers around the world have shown that smoking greatly impacts mental health and that it is associated with psychological disorders. However, little research exists on the relationship between the mental health and dyadic adjustment of smokers as compared to nonsmokers. One study at least (8) showed that among women, four psychosocial factors were associated with smoking: history of depression, increased marital conflict, greater number of undesirable life events, and full-time employment. Depression and marital conflict were also associated with higher alcohol-drinking levels. To help close the gap in the literature on the relationship between smokers’ mental health and dyadic adjustment, the present study attempts, with the input of the indigenous cultures of Sistan and Baluchestan, to answer the following questions:

1) Is there a significant relationship between the mental health and dyadic adjustment of smokers and nonsmokers?

2) Do the four sub-scales of mental health (physical symptoms, anxiety, social dysfunction, and depression) and the total scores of mental health predict the dyadic adjustment of smokers and nonsmokers?

3) Is there a significant difference between the mean scores of smokers and nonsmokers on a mental health scale and its sub-scales?

4) Is there a significant difference between the mean scores of smokers and nonsmokers on a dyadic adjustment scale?

## 2. Objectives

The study was performed to ascertain the relationship between mental health and dyadic adjustment of smokers and nonsmokers in Zahedan.

## 3. Patients and Methods

The population of this research consists of all smokers and nonsmokers in Zahedan City; 200 people (100 smokers and 100 nonsmokers) were selected by accessible sampling method for this study. The sample group age range was between 20 to 70 years old.

### 3.1. General Health Questionnaire

The 28-item General Health Questionnaire (GHQ-28) was developed by Goldberg (20) in order to identify psychotic mental disorders. The questionnaire measures the following four dimensions of mental health: physical symptoms, anxiety, social dysfunction, and depression. The GHQ-28 has been used by Cheung and Spears to diagnose minor psychological disorders in 1083 high school students in Hong Kong (21). The mean age of the sample was 15 years old. In that research, the Alfa co-efficient of physical symptoms was 0.67, anxiety 0.71, social dysfunction 0.59, and depression 0.75. In their study, Pourghaz and Tamini (22) found GHQ-28 reliability to be: physical symptoms 0.89, anxiety 0.76, social dysfunction 0.74, depression 0.83, and total mental health 0.91. The present study found Cronbach’s alpha coefficient for the four sub-scales and the entire questionnaire to be as follows: physical symptoms 0.86, anxiety 0.87, social dysfunction 0.60, depression 0.85, and overall GHQ 0.90.

### 3.2. Dyadic Adjustment Scale

The Dyadic Adjustment Scale (DAS) was developed by Spanier (23) to assess the level of dyadic adjustment of spouses. The DAS is a 32-item rating instrument written at an 8th-grade reading level; either or both partners in a relationship may complete it. Respondents are asked to rate each of the items on a Likert-type scale, choosing the most suitable response options. Respondents are also asked to indicate the extent of agreement or disagreement between the individual and his/her partner for each item. DAS includes the following four sub-scales: dyadic consensus, dyadic satisfaction, affectional expression, and dyadic cohesion. The most useful way of interpreting DAS is through the sub-scale scores, which are compared to norms for the appropriate group. The responses can be compared to couples not specifically identified as having a diagnosed problem (married normative group) or to individuals whose marriages were ended (divorced normative group). Lower scores on the DAS indicate a problem; higher scores indicate little or no problem. The reliability of the subscales are as follows: for dyadic satisfaction 0.94, for dyadic consensus 0.90, for dyadic cohesion 0.81, for affectional expression 0.73; the total reliability of this scale is 0.96. Molazadeh’s (24) study found the reliability of this scale, using Cronbach’s alpha, to be 0.86 and 0.89 for subscales relatively.

## 4. Results

“Is there a significant relationship between the mental health and dyadic adjustment of smokers and nonsmokers?”For the responses to this first research question, a Pearson correlation coefficient was conducted on the data, and the results are displayed in *Table 1*, which shows that dyadic adjustment has significant negative correlation with physical symptoms (r (200) = -0.467, *P* < 0.01); anxiety (r (200) = -0.398, *P* < 0.01); social dysfunction (r (200) = -0.269, *P* < 0.01); depression (r (200) = -0.191, *P* < 0.05); and overall GHQ scores (r (200) = -0.438, *P* < 0.01). The second research question was “Do the four sub-scales of mental health (physical symptoms, anxiety, social dysfunction, and depression) and the total scores of mental health predict the dyadic adjustment of smokers and nonsmokers?” Stepwise regression was applied to the response data to predict the dyadic adjustment from the mental health sub-scales and the overall GHQ scores. As shown in *Table 2*, the sub-scale of physical symptoms (β = -0.435, *P* < 0.001) was significantly related to dyadic adjustment, but social dysfunction (β = -0.141, *P* < 0.05) had significant negative relation with dyadic adjustment. The other mental health sub-scales of anxiety and depression, as well as the total GHQ, failed to enter into the regression equation; this shows that these scales were not significantly associated with dyadic adjustment. Physical symptoms accounted for 22.7% of the variance in dyadic adjustment; social dysfunction accounted for only 1.8% of the variance in dyadic adjustment. Obviously, the relation of social dysfunction to dyadic adjustment is not as great as that of physical symptoms. As shown in *Table 3*, social dysfunction (β = - 0.717, *P* < 0.001) has significant negative relation to dyadic adjustment. Too, social dysfunction (β = -1.017, *P* < 0.001) was associated negatively with dyadic adjustment. However, depression (β = 0.447, *P* < 0.01) and total GHQ scores (β = 0.652, *P* < 0.05) do have significant relation to dyadic adjustment. Physical symptoms failed to enter into the regression equation; this shows that the sub-scale of physical symptoms was not significantly associated with dyadic adjustment. Social dysfunction accounted for 30% of the variance in dyadic adjustment. In other words, social dysfunction is the greatest predictor for dyadic adjustment. In the second step, anxiety accounted for 3.7% variance in dyadic adjustment; in the third step, depression accounted for 7% variance in dyadic adjustment; and finally in the fourth step, overall GHQ accounted for 3.5% variance in dyadic adjustment. As shown in *Tabulation 1*, only the sub-scale of physical symptoms (β = -0.398, *P* < 0.001) has significant negative relation with dyadic adjustment. The other mental health sub-scales and the overall GHQ scores failed to enter into the regression equation; this shows that they were not significantly associated with dyadic adjustment. Finally for question two, physical symptoms accounted for 15.9% variance in dyadic adjustment.

**Table 1.** Pearson Correlation Coefficient Between Mental Health and Dyadic Adjustment

	Physical Symptoms	Anxiety	Social Dysfunction	Depression	Overall GHQ ^a^
Dyadic adjustment (n = 200)	-0.467 ^b^	-0.398 ^b^	-0.269 ^b^	-0.191 ^c^	-0.438 ^c^

^a^ Abbreviation: GHQ, general health questionnaire

^b^*P* < 0.01

^c^*P* < 0.05

**Table 2.** Stepwise Regression of Mental Health and Its Sub-Scale on Dyadic Adjustment in Whole Sample

	β	R ^a^	R Square	R Square Change	*P* value
Physical symptoms	-0.435	0.476	0.227	0.227	0.000
Social dysfunction	-0.141	0.495	0.245	0.018	0.031

^a^ Abbreviation: R, leaner relationship between two variables

**Table 3.** Stepwise Regression of Mental Health and Its Sub-Scale on Dyadic Adjustment in Smoker Sample

	β	R ^a^	R Square	R Square Change	*P* value
Social dysfunction	-0.717	0.548	0.30	0.30	0.000
Anxiety	-1.017	0.581	0.337	0.037	0.000
Depression	0.447	0.638	0.442	0.07	0.008
Overall GHQ ^a^	0.652	0.655	0.442	0.035	0.016

^a^ Abbreviations: GHQ, general health questionnaire; R, leaner relationship between two variables

**Tabulation 1.** Stepwise Regression of Mental Health and its Sub-Scale on Dyadic Adjustment in None-Smoker Sample

	β	R ^a^	R Square	*P* value
Physical symptoms	-0.398	0.398	0.159	0.000

^a^ Abbreviation: R, leaner relationship between two variables

“Is there a significant difference between the mean scores of smokers and nonsmokers on a mental health scale and its sub-scales?” For this third research question, an independent sample t-test was conducted on the response data to compare the mental health and its sub-scales of smokers and nonsmokers. As displayed in *Table 4*, results show significant differences in scores for smokers and nonsmokers on physical symptoms (t = 6.398, P < 0.001), anxiety (t = 2.425, P < 0.05), and overall scores of GHQ (t = 3.100, P < 0.01). Smokers showed higher mean scores on these sub-scales in comparison to nonsmokers, but the results did not reveal any significant difference between the two groups on the sub-scales of social dysfunction and depression. The fourth and final research question was, “Is there a significant difference between the mean scores of smokers and nonsmokers on a dyadic adjustment scale?” An independent sample t-test was conducted on the response data to compare the dyadic adjustment for smokers and nonsmokers. The results, in *Table 5*, reveal a significant difference in scores for smokers and nonsmokers on dyadic adjustment (*t* = -14.001, *P* < 0.001). Nonsmokers obtained higher mean scores in comparison to smokers.

**Table 4.** Results of Independent “t” test Between Smokers and Nonsmokers on GHQ and Its Sub-Scales

	Mean ± SD	*t*-Test	df	*P* value
**Physical symptoms**		6.398	198	0.000
Smokers	5.0800 ± 4.03189			
Nonsmokers	1.7500 ± 3.29179			
**Anxiety**		2.425	198	0.016
Smokers	4.9400 ± 3.71407			
Nonsmokers	3.3800 ± 5.25276			
**Social dysfunction**		1.838	198	0.068
Smokers	6.2600 ± 2.95290			
Nonsmokers	5.3800 ± 3.76797			
**Depression**		-0.829	198	0.408
Smokers	2.7400 ± 3.31394			
Nonsmokers	3.2000 ± 4.44949			
**Overall GHQ ** **^a^**		3.100	198	0.002
Smokers	19.0200 ± 11.47415			
Nonsmokers	13.7100 ± 12.71704			

^a^ Abbreviation: GHQ, general health questionnaire

**Table 5.** Results of Independent “t” test Between Smokers and Nonsmokers on Dyadic Adjustment

	Mean ± SD	*t*-Test	df	*P* value
**Dyadic adjustment (n = 200)**		-14.001	98	0.000
Smokers	9.5700 ± 14.34936			
Nonsmokers	125.3600 ± 11.55008			

## 5. Discussion

In the human condition, some tendencies join with psychological disorders to negatively impact individuals’ social and personal lives. Dependency on cigarettes is one of them; financial expenditures for cigarettes and negative social attitudes towards their potential harm may create family problems and imperil a family’s mental health status. Contemporary families worry that smoking will lead to drug abuse. Families know that smoking may cause other problems as well, for instance, common affective disorders that negatively impact children’s ability to learn or serious and eventually fatal diseases smokers’ physical health. The results from the first research question showed that mental health and its sub-scales (physical symptoms, anxiety, social dysfunction, and depression) had significant negative correlation with dyadic adjustment. In other words, increased scores of mental health indicate decreased dyadic adjustment levels; conversely, decreased scores of mental health indicate increased dyadic adjustment levels. We should consider that higher mean scores on the GHQ-28 indicate a lower mental health state and psychological disorders. Few studies have been conducted on the relationship of mental health and dyadic adjustment in smokers. A study (24) by Cohen et al. (8) showed that depression and marital conflict were associated with higher alcohol-drinking levels. The results of the second research question showed that physical symptoms accounted for 22.7% of variance in dyadic adjustment, and in the second step, social dysfunction accounted for 1.8% of variance in dyadic adjustment in the whole sample. Other sub-scales of mental health failed to enter into the equation regression, indicating they were not associated with dyadic adjustment. Physical symptoms had the greatest relationship with dyadic adjustment, and the sub-scale of physical symptoms was a stronger predictor for dyadic adjustment than was social dysfunction. However, in the smokers ’sample, social dysfunction accounted for 30% of the variance in dyadic adjustment; in other words, social dysfunction was the greatest predictor for dyadic adjustment. In the second step, anxiety accounted for 3.7% of variance in dyadic adjustment; in the third step, depression accounted for 7% of variance in dyadic adjustment; and finally in the fourth step, overall GHQ accounted for 3.5% of variance in dyadic adjustment. But the depression sub-scale failed to enter into the equation regression, demonstrating that it was not associated with dyadic adjustment. Eventually, the results of stepwise regression illustrated that only physical symptoms accounted for 15.9% of variance in dyadic adjustment; thus, it was the strongest predictor for dyadic adjustment. Other sub-scales of mental health failed to enter into the equation regression. There is not enough research about mental health and dyadic adjustment in the smokers’ sample to know whether the mental health dimensions are associated with dyadic adjustment or whether these dimensions could predict dyadic adjustment. The results from the third research question revealed a significant difference in scores for smokers and nonsmokers on physical symptoms, anxiety, and overall scores of mental health. Smokers exhibited higher mean scores on these sub-scales in comparison to nonsmokers. The results, however, did not reveal significant differences between the two groups on social dysfunction and depression. The results of this study conform to the research results of Dudas (11). These investigators found that students who smoked had higher mean scores on anxiety symptoms. Their results also showed that students who smoked for fun exhibited higher anxiety than nonsmokers. Other researchers have reported similar results (9, 12-18): smoking negatively impacts mental health, and smokers exhibit lower mental health states than nonsmokers. At the same time, smoking impacts physical health, decreases mental health, and creates challenges for the smoker and his/her family. The results of the fourth research question demonstrated a significant difference between smokers and nonsmokers, i.e., smokers showed poorer dyadic adjustment than nonsmokers. This study’s results conform also to those of Cohen, et al. (8). Individuals who do not smoke display better dyadic adjustment; nonsmokers show greater dyadic consensus, dyadic satisfaction, affectional expression, and dyadic cohesion than smokers. To sum up, smokers experience more marital adjustments. Thus it can be deduced that smoking imperils familial harmony and simultaneously threatens couples’ dyadic adjustment.
